# Non-Hematologic Toxicity of Bortezomib in Multiple Myeloma: The Neuromuscular and Cardiovascular Adverse Effects

**DOI:** 10.3390/cancers12092540

**Published:** 2020-09-07

**Authors:** Elia Pancheri, Valeria Guglielmi, Grzegorz M. Wilczynski, Manuela Malatesta, Paola Tonin, Giuliano Tomelleri, Dominika Nowis, Gaetano Vattemi

**Affiliations:** 1 Department of Neurosciences, Biomedicine and Movement Sciences, Section of Clinical Neurology, University of Verona, 37134 Verona, Italy; elia.pancheri@univr.it (E.P.); valeria.guglielmi@univr.it (V.G.); paola.tonin@aovr.veneto.it (P.T.); giuliano.tomelleri@univr.it (G.T.); 2 Laboratory of Molecular and Systemic Neuromorphology, Department of Neurophysiology Warsaw, Nencki Institute of Experimental Biology, 02-093 Warsaw, Poland; g.wilczynski@nencki.gov.pl; 3 Department of Neurosciences, Biomedicine and Movement Sciences, Section of Anatomy and Histology, University of Verona, 37134 Verona, Italy; manuela.malatesta@univr.it; 4 Department of Immunology, Medical University of Warsaw, 02-093 Warsaw, Poland; dnowis@wum.edu.pl; 5 Laboratory of Experimental Medicine, Medical University of Warsaw, 02-093 Warsaw, Poland

**Keywords:** multiple myeloma, proteasome inhibitors, bortezomib, peripheral neuropathy, cardiotoxicity, muscle toxicity

## Abstract

**Simple Summary:**

Multiple myeloma (MM) is a still uncurable tumor of mainly elderly patients originating from the terminally differentiated B cells. Introduction to the treatment of MM patients of a new class of drugs called proteasome inhibitors (bortezomib followed by carfilzomib and ixazomib) significantly improved disease control. Proteasome inhibitors interfere with the major mechanism of protein degradation in a cell leading to the severe imbalance in the protein turnover that is deadly to MM cells. Currently, these drugs are the mainstream of MM therapy but are also associated with an increased rate of the injuries to multiple organs and tissues. In this review, we summarize the current knowledge on the molecular mechanisms of the first-in-class proteasome inhibitor bortezomib-induced disturbances in the function of peripheral nerves and cardiac and skeletal muscle.

**Abstract:**

The overall approach to the treatment of multiple myeloma (MM) has undergone several changes during the past decade. and proteasome inhibitors (PIs) including bortezomib, carfilzomib, and ixazomib have considerably improved the outcomes in affected patients. The first-in-class selective PI bortezomib has been initially approved for the refractory forms of the disease but has now become, in combination with other drugs, the backbone of the frontline therapy for newly diagnosed MM patients, as well as in the maintenance therapy and relapsed/refractory setting. Despite being among the most widely used and highly effective agents for MM, bortezomib can induce adverse events that potentially lead to early discontinuation of the therapy with negative effects on the quality of life and outcome of the patients. Although peripheral neuropathy and myelosuppression have been recognized as the most relevant bortezomib-related adverse effects, cardiac and skeletal muscle toxicities are relatively common in MM treated patients, but they have received much less attention. Here we review the neuromuscular and cardiovascular side effects of bortezomib. focusing on the molecular mechanisms underlying its toxicity. We also discuss our preliminary data on the effects of bortezomib on skeletal muscle tissue in mice receiving the drug.

## 1. Introduction

Multiple myeloma (MM) is a neoplastic disorder characterized by clonal proliferation of malignant plasma cells in the bone marrow, monoclonal protein in the blood and/or urine, and evidence of specific end-organ damage including hypercalcemia, renal failure, anemia, and osteolytic bone lesions [1,2]. The disease is almost always preceded by an asymptomatic premalignant stage termed monoclonal gammopathy of undetermined significance (MGUS), with or without an identified intervening stage of smoldering multiple myeloma (SMM) [3,4].

MM is the second most common hematological malignancy in adults and accounts for about 1.8% of all cancers and 10% of all blood ones in the United States with an annual incidence of 7.0 per 100,000 [4]. It affects mainly the older population with a median age of patients at diagnosis of 69 years, is more common in men and among people of African American descent [5]. Despite the significant progress in available therapies and the improvement in the outcome of patients, MM remains an incurable disease with a 5-year relative survival rate of 53.9% [5,6,7].

The overall approach to MM treatment has dramatically changed over the last two decades. New classes of drugs have been added to the traditional armamentarium (corticosteroids, alkylating agents, and anthracyclines), and, along with high-dose chemotherapy followed by autologous hemopoietic stem cell transplantation, have led to a longer-lasting clinical response in these patients. Novel agents include proteasome inhibitors, immunomodulatory drugs (thalidomide, lenalidomide, pomalidomide), histone deacetylase inhibitors (panobinostat), monoclonal antibodies directed against CD38 (daratumumab and isatuximab) or signaling lymphocytic activation molecule F7 (SLAMF7, elotuzumab), and nuclear export inhibitors (selinexor) [8,9]. Currently, MM therapy is based on regimens that combine agents having different mechanisms of action.

Proteasome inhibitors (PIs) have been proved to be effective in MM patients and include the first-in-class PI bortezomib, the second-generation inhibitor carfilzomib, and ixazomib, the first oral PI [10]. These drugs target the 26S proteasome, a multi-subunit ATP-dependent enzymatic complex of the ubiquitin-proteasome system (UPS), the major protein degradation pathway in eukaryotic cells [10]. The proteasome is made up of a barrel-shaped core particle consisting of four stacked heptameric rings, referred to as the 20S proteasome, and two 19S regulatory particles at the extremities [10]. Proteasome substrates include misfolded, damaged, or mutant proteins and short-lived regulatory proteins that need to be removed for proper cellular function [10]. The accumulation of aberrantly folded proteins in the endoplasmic reticulum (ER) lumen can affect ER homeostasis and lead to a condition referred to as ER stress [10]. One pathway of relieving ER stress is the ER-associated degradation (ERAD) by which unfolded/misfolded proteins undergo retrotranslocation to the cytosol and are degraded by UPS [11,12]. By inhibiting the 26S proteasome, PIs halt ERAD and cause protein overload within the ER [11,12]. MM cells, because of their high protein turnover due to immunoglobulin overproduction, are particularly susceptible to proteasome inhibition [13]. Other proven mechanisms of anti-myeloma activity of PIs include the induction of oxidative stress due to excessive production of reactive oxygen species, which further exacerbates protein misfolding but also damages DNA, inevitably leading to cell death [14]. 

## 2. Bortezomib: The First-in-Class Proteasome Inhibitor 

The introduction of bortezomib (also known as PS 341 and Velcade^™^), approved by the US Food and Drug Administration (FDA) in 2003, represented a breakthrough in the treatment of MM. The drug is currently indicated for both newly diagnosed and relapsed and/or refractory forms of MM [15,16] and can be used in combination with other agents in stem cell-eligible patients, both in induction and in consolidation or maintenance therapy, as well as in patients not eligible for stem cell transplantation [8,17]. Bortezomib is a reversible boronic acid dipeptide which binds primarily the β5 subunit and, to a lesser extent, the β2 and β1 subunits of the catalytic 20S proteasome, blocking its chymotrypsin-like, trypsin-like and caspase-like activities, respectively [18,19]. Evidence that point mutations in the gene encoding the proteasome β5 subunit (*PSMB5*) result in bortezomib resistance suggests that this subunit is the clinically relevant target of the drug [20,21,22].

**Figure 1:**
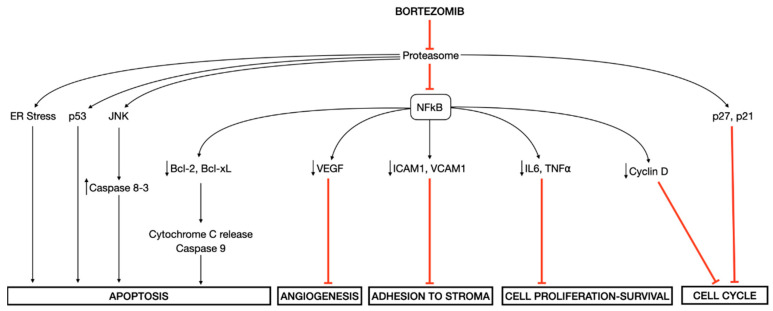
Molecular mechanisms of action of bortezomib. Bortezomib induces several downstream effects, among which the endoplasmic reticulum (ER) stress and the inhibition of transcription factor nuclear factor-κB (NF-κB) play a central role in mediating its cellular toxicity.

Besides inducing ER stress in MM cells [23,24], bortezomib prevents the degradation of an inhibitory molecule IκB with the consequent suppression of transcription factor nuclear factor-κB (NF-κB) pathway, which regulates the expression of genes involved in cell growth and survival, cell cycle regulation, angiogenesis, inflammation, interactions between tumor cells and bone marrow stromal cells, and anti-apoptotic mechanisms [25]. Other proposed mechanisms of cellular toxicity induced by bortezomib are the direct induction of caspase-mediated apoptosis via the activation of the c-Jun NH2-terminal kinase (JNK) pathway and the phosphorylation of p53, and the cell-cycle G1 arrest via increased levels of cyclin-dependent kinase inhibitors p27 and p21 [26,27] (Figure 1). 

**Figure 2:**
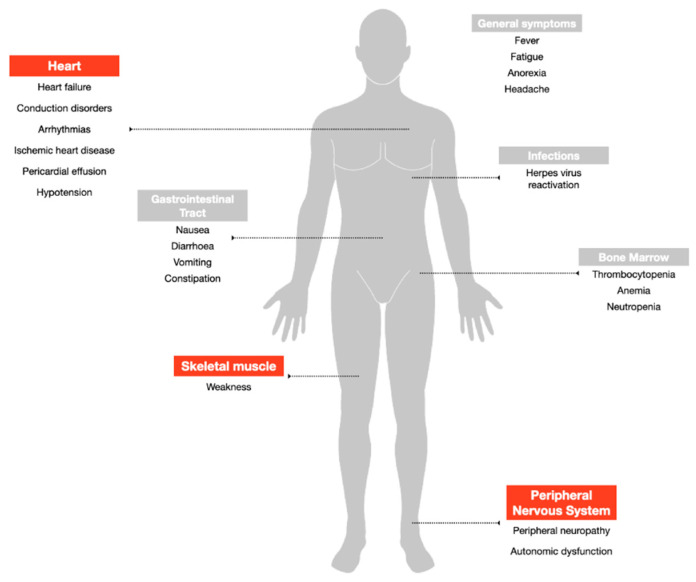
Adverse events associated with bortezomib treatment. Highlighted in red the side effects addressed in the review.

Despite being among the most widely used and highly effective treatment options for MM, bortezomib can induce adverse events that potentially lead to early discontinuation of the therapy with a negative impact on the quality of life and outcome of the patients (Figure 2). The drug is available for intravenous injection or subcutaneous use [28]. Based on data from the multicenter phase II open-label, single-arm (SUMMIT) trial, which led to the FDA approval of the drug, the most frequently reported all-grade side effects were asthenic conditions including fatigue and weakness (52%), nausea (55%), diarrhea (44%), thrombocytopenia (40%), peripheral neuropathy (31%), vomiting (27%), anorexia (25%), pyrexia (22%) and anemia (21%) [29]. The major grade 3 adverse reactions included thrombocytopenia (28%), fatigue (12%), neuropathy (12%), and neutropenia (11%), and fourteen percent of the patients experienced at least one episode of severe toxicity (grade 4), most commonly hematologic including thrombocytopenia (3%) and neutropenia (3%) [29]. The major adverse effects reported in the following APEX phase III trial were consistent in type and frequency with those previously described [30].

Among non-hematological toxicities of bortezomib in MM treated patients, peripheral neuropathy received close attention while cardiac and skeletal muscle adverse events have been little considered. This review provides a detailed overview of the neuromuscular and cardiovascular unwanted effects of bortezomib. We focus on the molecular mechanisms responsible for drug toxicity in these tissues, summarizing the key data from clinical studies and from in vivo and in vitro experimental models. 

## 3. Peripheral Neuropathy: The Key Dose-Limiting Toxicity 

Peripheral neuropathy represents one of the most common, unpredictable, and dose-limiting non-hematologic adverse events of MM treatment with bortezomib, which often requires dose modification, delay, or discontinuation of the drug. Bortezomib-induced peripheral neuropathy (BIPN) negatively affects clinical endpoints with a detrimental effect on the quality of life of patients and is associated with a significant economic impact, which adds up to the costs directly related to disease therapy [31,32].

BIPN is a length-dependent predominant sensory painful neuropathy with the potential involvement of both large and small nerve fibers. It is clinically characterized by paraesthesia and numbness with symmetric “glove and stocking” distribution, and by neuropathic pain, mostly affecting fingertips and toes [31]. Neurological examination reveals distal multimodal sensory loss and reduced or absent deep tendon reflexes while peripheral nerve motor impairment is usually subclinical [33]. Symptoms of autonomic dysfunction, including orthostatic hypotension, postural dizziness, syncope, diarrhea, paralytic ileus, and urinary disturbances have been reported [34,35,36] and are likely due to bortezomib toxicity of the pre- and post-ganglionic autonomic nerve fibers of the sympathetic and parasympathetic nervous system [34,37,38]. Nerve conduction studies reveal a predominantly axonal or mixed polyneuropathy, although primarily demyelinating features have been occasionally reported [39,40,41,42,43]. Needle electromyography may record neurogenic motor unit potentials with active denervation in the distal muscles of the lower extremities [31]. Usually, cerebrospinal fluid analysis is not informative [31]. In a handful of individual case series or single case reports, bortezomib was associated with prominent and progressive motor neuropathy [38,40,41,43,44,45,46,47]. This condition has been ascribed to an immune-mediated mechanism for the frequent finding of albumin-cytological dissociation in the cerebrospinal fluid and for the favorable response to corticosteroids, intravenous immunoglobulin, or plasma exchange [38,40,41,43,45,46,47]. Moreover, in a few patients, a vasculitis process has been observed on nerve pathological examination [38,47]. 

A retrospective analysis of 8218 MM patients included in phase 3 randomized control trials with bortezomib reported an overall incidence of peripheral neuropathy (all grades) ranging from 8.4% to 80.5% (median 37.8%) and of severe neuropathy (grades 3 and 4) ranging from 1% to 33.2% (median 8%) [48]. The incidence and the severity of the neuropathy were similar for both newly diagnosed and relapsed MM patients [48,49]. Due to neurotoxicity, the drug was reduced in 12% and discontinued in 5% of the patients [50].

The incidence and severity of peripheral neuropathy depend on the cumulative drug dose, the dosing schedule, and the route of administration. Neurotoxicity generally develops within the first cycles of treatment using the standard dose and schedule, and reaches a plateau after five 3-week cycles at a cumulative dose of 42 mg/m^2^ in relapsed MM or after three 6-week cycles at a cumulative dose of 45 mg/m^2^ in newly diagnosed MM [33,50]. There is no evidence of new cumulative neurotoxicity upon retreatment with bortezomib [51,52]. The once per week and the subcutaneous bortezomib administration are associated with a significantly lower rate of neuropathy compared to the twice per week regimen and the intravenous route, without efficacy reduction [49,53,54,55]. Despite its frequency and severity, BIPN presents a potentially favorable outcome. Dose reduction, modification in the route or schedule of administration, and ultimately withdrawal of therapy can improve and sometimes resolve the neuropathy [56,57]. The median time to recovery is approximately three months [49,58,59]. However, neurotoxicity can be an adverse event that continues to affect patients after treatment has ceased, and up to 30% of patients may be left with persistent neuropathy [33,60]. BIPN is not preventable, and the treatment approach is limited to symptomatic therapies aiming at reducing neuropathic pain [61]. Immune therapy should be considered if there is any evidence of immune-mediated neuropathy.

Possible predisposing factors for the development of the treatment-emergent neuropathy have been identified. A pre-existing neuropathy significantly increases the risk of developing BIPN [31,58,62,63]. The peripheral nerve involvement may depend on previous chemotherapy or can be related to the hematologic disease itself [49]. Clinical signs of peripheral neuropathy or subclinical neurophysiological abnormalities have been observed in up to 54% of patients with previously untreated symptomatic MM [64]. It is not clear if the combination of thalidomide, a neurotoxic agent, and bortezomib results in a higher susceptibility to peripheral nerve toxicity [48]. Advanced age has been proposed as a risk factor for drug-induced peripheral neuropathy [65,66]; however, this relationship was not confirmed in larger trials [50,58] suggesting that elderly patients without any significant comorbidities should be treated with the optimal bortezomib dose [33]. Diabetes and impairment of renal function have been ruled out as predisposing factors for neuropathy [49]. Intriguingly, neurotoxicity is more frequent in patients with MM than in those with other malignancies, suggesting that the disease itself may have a role in the development of neuropathy in bortezomib-treated patients [49]. 

The pathogenesis of BIPN is not yet fully elucidated. In vivo and in vitro experimental studies have been designed to characterize the pathological features of the neuropathy and to understand the mechanisms through which bortezomib exerts its toxicity on the peripheral nervous system. 

Due to the absence of the blood-brain barrier that bortezomib cannot cross, peripheral axons and dorsal root ganglia (DRG) of primary sensory neurons are selectively vulnerable, whereas the motoneurons in the anterior horn of the spinal cord are usually spared [67,68]. As mentioned above, bortezomib-treated patients, in most cases, present a sensory neuropathy and neuropathic pain with impairment of afferent large myelinated Aβ fibers as well as of small, thinly myelinated Aδ and unmyelinated C fibers [69,70]. Severely reduced autonomic nerve fiber densities were also observed in skin biopsies of treated patients suggesting that bortezomib can cause neuropathy involving somatic as well as autonomic small fibers with a length-dependent pattern [34]. 

Animal models for BIPN reproduce the findings observed in the clinical setting. Neuropathological examination of peripheral nerves from adult rats exposed to bortezomib three times per week for 8 weeks or twice per week for 4 weeks and from mice receiving bortezomib twice per week for 6 weeks documented morphological abnormalities and/or reduction of myelinated and unmyelinated fibers with possible concurrent impairment of DRG of sensory neurons and satellite cells [68,71,72,73,74]. Pathological changes in sensory nerves consisted of vacuolization and disorganization of fiber axoplasm [71]. Alterations of Schwann cells and myelin sheaths have also been described in both rat and mice models, although bortezomib mainly induces an axonopathy without significant impairment of internodal myelin structure [75,76,77]. As already mentioned, in rare cases, bortezomib can also induce neuropathy with predominant demyelinating features based on electrodiagnostic and histopathological findings [39,40,41,47].

Different hypotheses have been raised on the molecular mechanisms underlying these alterations. It has been suggested that mitochondria are one of the main targets of bortezomib in the peripheral nervous system. In the rat model of BIPN, swollen and enlarged mitochondria were observed in axons, Schwann cells, DRG satellite cells, and occasionally in DRG neurons, associated with a dysfunction in mitochondrial respiration with reduced complex I and II enzyme activities and ATP production [76,78,79]. Impaired mitochondrial function can induce the formation of reactive oxygen species and, as in MM cells [80], a dysregulation of intracellular Ca2+ homeostasis, both mechanisms being potentially neurotoxic [81]. Moreover, reduced ATP production could impair the function of the Na+-K+-ATPase-dependent pump with early negative effects on axonal membrane excitability, thus contributing to the neuropathic pain [82]. Despite the mechanisms leading to mitochondrial defects remain unknown, their dysfunction is considered an indirect effect of cytosolic proteasome inhibition rather than an off-target effect of bortezomib [78,79]. The “mitotoxicity hypothesis” has been proposed also for other chemotherapeutic agents, such as taxanes and platinum agents [83,84].

It has been shown that some drugs act on microtubule depolymerization and polymerization with the consequent disruption of processes necessary for cell survival, including intracellular transport, cell motility, maintenance of cell structure, and mitosis [85]. In vitro studies showed impaired axonal growth and axonal degeneration in neonatal and adult primary neuron cultures treated with MG132 and lactacystin, two other 26S proteasome inhibitors [86,87]. Bortezomib has been demonstrated to induce increased tubulin polymerization and stabilization of microtubules in neuronal cell lines as well as in rat embryonic and adult DRG neurons [88,89]. The mechanism promoting tubulin polymerization does not derive from a direct microtubule-binding as occurs for paclitaxel [85], but it is probably due to a selectively increased level of MAP2, a microtubule-associated protein [88] as a result of proteasome inhibition. The altered microtubule dynamics result in decreased neurite elongation and impaired axonal transport of mitochondria in mice and rat DRG neuronal cultures, respectively [90,91]. In addition, bortezomib can affect another major component of the cytoskeleton, the actin filaments, as observed in mouse neural stem cells [92].

Besides the effects on mitochondria and cytoskeleton, bortezomib acts at the nuclear level in primary sensory neurons, without affecting their survival [67,93]. In DRG neurons of adult Sprague-Dawley rats treated with low doses of bortezomib, nuclei were displaced in an eccentric position and resulted in irregular shape and altered polarity [93]. Ultrastructural analysis of DRG neurons documented a partial chromatolysis with dissolution of Nissl Bodies and retention of polyadenylated RNAs in nuclear granules, suggesting that bortezomib could interfere with mRNA processing, thus causing impairment of protein synthesis [67]. In support of this, reduced expression of the brain-derived neurotrophic factor (BDNF), a neuronal growth factor that is crucial for neuronal survival and repair, was observed in dysfunctional neurons [93]. 

Recently, a dysregulation of the sphingolipid metabolism, which may contribute to the development of neuropathic pain was demonstrated in the dorsal horn of the spinal cord from rats exposed to bortezomib [94]. These findings highlight that bortezomib affects different intracellular processes.

Several clinical and histopathological data suggest that also inflammation might be a relevant factor in the onset and course of BIPN [38,40,47]. A small-sized multicentre phase II trial reported a shift in the T helper type 1 (Th1)/ T helper type 2 (Th2) cytokine balance towards Th2 dominance with elevated levels of interleukin-6 (IL-6) in the peripheral blood of patients who developed a painful neuropathy during treatment with bortezomib [95]. The role of immunity in bortezomib-induced neurotoxicity, however, remains controversial since mice immunocompromised by X-ray irradiation before bortezomib treatment displayed the same peripheral neuropathy of immunocompetent mice [72]. Conversely, in animal models of bortezomib-induced neuropathy, the increased expression of TNF-α, a proinflammatory cytokine associated with the development of neuropathic pain [96,97,98,99], has been observed in the neurons of DRG and spinal dorsal horn [100,101,102]. Blockade of TNF-α signaling prevents the loss of myelinated and unmyelinated peripheral nerve fibers [100] and can hamper the onset of neuropathic pain in animal models for this condition [101,102,103,104]. One of the main mechanisms of action of bortezomib in cancer cells is the inhibition of the NF-*κ*B pathway by blocking the proteasome degradation of its negative regulator IkB with consequently reduced production of proinflammatory cytokines including TNF-α [105,106]. In contrast to cancer cells, bortezomib seems to activate NF-*κ*B in DRG neurons of treated mice independently of the presence of its natural inhibitor, thus leading to an upregulation of proinflammatory cytokines [100]. Indeed, neuropathy is significantly less severe in mice with impaired NF-*κ*B activation than in wild-type animals [107].

## 4. Cardiovascular Toxicity: The Low Rate and Reversible Side Effect

There are ambiguous and conflicting data on the influence of bortezomib on cardiac function and its potential cardiotoxicity is still a matter of debate. This partly depends on whether the drug is used in patients with significant cardiovascular disease risk factors and on previous exposure to known cardiotoxic chemotherapeutic agents such as anthracyclines, making it difficult to determine if a single or a combination of factors cause the cardiac events [108,109]. However, since bortezomib has been approved to be used in the clinical setting, many reports have been published addressing its cardiovascular toxicity [110,111,112,113,114,115,116,117,118,119,120,121,122,123,124,125,126]. The cardiovascular adverse events so far associated with bortezomib treatment include heart failure, that is the most frequently reported cardiac side effect [110,111,112,113,114,115,116,117,118,119,120], conduction disorders such as complete atrioventricular block [110,121,122,123], arrhythmias including atrial fibrillation [110,124], ischemic heart disease [125], pericardial effusion [112,126] and orthostatic hypotension [27,115]. 

A systematic review and meta-analysis of 25 prospective phase II and phase III trials evaluating bortezomib in the treatment of different malignancies including MM, lymphoma, Waldenström macroglobulinemia, non-small cell lung cancer, and ovarian cancer concluded that it does not significantly increase the risk of cardiac adverse events as compared to control medications, with an incidence of all grade and high-grade cardiotoxicity in all patients of 3.8% and 2.3% respectively [127]. The overall cardiac safety profile of bortezomib was confirmed in a later retrospective analysis of patients included in the phase 2 registration study for the US and EU regulatory approval and in all phase 3 studies that led to the US and EU regulatory approval [128]. The study showed no significant differences in the incidence of cardiovascular toxicities between bortezomib- and non-bortezomib based arms. The incidence of grade ≥3 heart failure was 1.2–4.7%, with a low incidence of grade ≥3 ischemic heart disease (0.4–2.7%), arrhythmias (0.6–4.1%), and cardiac death (0–1.4%) [128]. In a recent prospective observational study, 17% of 30 patients with relapsed MM receiving bortezomib manifested cardiovascular adverse events confirming a low rate of cardiac toxicity of this drug compared to carfilzomib [129]. Cardiac complications usually occur within the first three months of treatment and mostly in patients with multiple cardiovascular risk factors, raising the possibility of a synergistic effect [129,130]. Cardiovascular side effects often improve with the withdrawal of the therapy, but medication, hospitalization, and pacemaker implantation may be required [110,112,115].

In the heart, the ubiquitin-proteasome system (UPS) is important in different intracellular pathways and its dysfunction has been suggested to play a pathogenetic role in cardiac diseases including cardiomyopathies, heart failure, ischemia-reperfusion injury, and atherosclerosis [131,132,133,134]. In vitro studies demonstrated that bortezomib at submicromolar concentrations inhibits reversibly the chymotrypsin-like proteasomal activity also in primary neonatal rat cardiomyocytes besides cancer cells [135,136]. Hence, it is reasonable to suppose that the drug could induce cardiac damage. 

Cardiovascular effects of bortezomib have been addressed in several in vivo studies that led to contradictory results. Left ventricular systolic and diastolic function was preserved and no distinct morphological myocardial abnormalities were detected in adult male rabbits exposed to bortezomib alone for 10 weeks [136]. Conversely, male Wistar rats treated thrice weekly with bortezomib for 3 weeks developed a reversible cardiac dysfunction with a significant decrease in left ventricular ejection fraction and showed myocardial changes with scattered enlarged and vacuolated cardiomyocytes [137]. Bortezomib treatment induced left ventricular hypertrophy in 10-week-old mice subjected to sham surgery and resulted in heart failure and premature death in animals with aortic constriction [138,139]. Interestingly, three-month-old female pigs exposed for 12 weeks to MLN273, a proteasome inhibitor structurally and functionally related to bortezomib [140,141,142], developed hypertrophic-restrictive cardiomyopathy with a significant increase of myocardial perivascular and interstitial fibrosis [143]. 

The pathogenic mechanisms potentially involved in bortezomib-induced cardiovascular toxicity remain largely to be elucidated. Myocardial cells are post-mitotic terminally differentiated cells with finely tuned metabolism and function, and limited capacity for division and proliferation. Quality control of structural and functional proteins and efficient removal of damaged cellular components play a key role in maintaining myocardial viability and regulating its functions [144]. In rat cardiomyoblast H9c2 cells, bortezomib, by inhibiting proteasomal activity, causes the accumulation of polyubiquitinated proteins which, in turn, leads to ER stress and compensatory autophagy [137]. MG262, a boronic acid-based proteasome inhibitor [142], through the activation of the calcineurin-NFAT pathway, promotes the translocation of the nuclear factor of activated T-cells (NFAT) into the nucleus in neonatal rat ventricular myocytes with significant changes in the cell morphology [138]. It has also been suggested that the inhibition of the proteasome by bortezomib in primary neonatal rat ventricular myocytes can lead to the activation of caspase-3 and caspase-7 and, consequently, to cellular apoptosis [135]. Mitochondria have been identified as another possible intracellular, and probably indirect target of cardiotoxicity because bortezomib inhibits complex V of the mitochondrial respiratory chain that results in a marked drop in ATP synthesis in the heart of treated rats and a decreased cell shortening of primary rat left ventricular myocytes [137]. Functional changes paralleled the structural alterations of the mitochondria, which became pleomorphic and enlarged containing concentric cristae and electron-dense inclusions and with a tendency to form large intermyofibrillar clusters leading to misalignment of the myofibrillar network [137]. Both the functional and morphological changes in this rat model of cardiotoxicity were reversible with full recovery after bortezomib discontinuation, suggesting that it does not induce irreversible damage of cardiomyocytes, in agreement with the improvement of heart function observed in MM patients after drug withdrawal [137]. Moreover, bortezomib-mediated mitochondrial dysfunction might be further explained by a recently described process of extraction of misfolded proteins from mitochondria, and their subsequent degradation in proteasomes altogether called mitochondria-associated degradation (MAD) [145]. Similarly to ERAD, inhibition of proteasome leads to accumulation of misfolded and damaged proteins in mitochondria resulting in their dysfunction.

It has been proposed that bortezomib-induced vascular effects could be partly responsible for its potential cardiotoxicity. Thrombotic microangiopathy has been recently described in patients treated with bortezomib, suggesting its possible direct microvascular toxicity to the endothelium [146]. In vitro studies demonstrated that bortezomib at clinically achievable concentrations exerts anti-angiogenic effects on endothelial cells derived from the bone marrow of MM patients and human umbilical vascular endothelial cells (HUVEC) [147]. Bortezomib mediates anti-angiogenesis both directly by inducing cell cycle arrest at G2 to M transition and indirectly through the down-regulation of the expression of genes involved in the angiogenic cascade, including VEGF and IL-6, with consequent growth inhibition and increased permeability of vascular endothelial cells [147,148]. The reversible peptide aldehyde MG132 [142] reduces in vitro the number of endothelial progenitor cells and negatively affects their function by inducing their apoptosis and impairing the endothelial nitric oxide synthase/nitric oxide (eNOS/NO) pathway [149,150]. Moreover, in female pigs, the chronic inhibition of proteasome by MLN273 can lead to endothelial dysfunction and coronary atherosclerosis with reduced myocardial perfusion response and increased microvascular permeability [141,143]. These data are supported by human studies that showed decreased proteasomal activity and accumulation of ubiquitinated substrates in carotid and coronary symptomatic unstable atherosclerotic plaques [151,152]. Therefore, bortezomib could affect the physiological properties of blood vessels and hasten or aggravate an already present vascular damage, as it happens in coronary heart disease.

Finally, the impairment of both the sympathetic and parasympathetic nerve fibers [34,37] could contribute to the conduction disorders and arrhythmias occasionally reported and participate in the postural hypotension observed in bortezomib treated patients [27].

## 5. Muscle Toxicity: The Neglected Adverse Event 

Patients with MM can develop several neurological complications caused either by the disease itself or by the toxic effects of the medications used to treat it. The involvement of skeletal muscle is considered very rare in MM and has been reported in the concurrent immunoglobulin light chain (AL) amyloidosis or as the result of long-lasting corticosteroid therapy [153]. Based on initial data from the phase II SUMMIT and CREST studies in relapsed and/or refractory MM, muscle weakness is a frequent adverse effect of bortezomib therapy, with the incidence of any grade and grade 3 events in patients of 11% and 4–5%, respectively [29,154]. Despite these elevated rates of weakness in patients, a detailed description and evaluation of skeletal muscle involvement with bortezomib was lacking in the literature. Recently, a small-sized single-center prospective study on 24 newly diagnosed symptomatic MM patients pointed out that bortezomib treatment may cause a myopathy [155]. Seven out of 14 patients receiving bortezomib complained of difficulty in climbing stairs and rising from a chair and developed a symmetrical proximal lower limb muscle weakness involving predominantly thigh flexors. All patients had serum creatine kinase (CK) within the normal reference range. Nerve conduction studies were normal, and needle electromyography (EMG) recorded myopathic features including small, polyphasic units, and fast recruitment. Bortezomib-induced myopathy was an early complication occurring within the four 3-week cycles of therapy and did not progress, remaining confined to the legs. Muscle weakness was potentially reversible, with 43% of patients experiencing symptom resolution in a median of 3.6 months with drug interruption. The mechanisms underlying the pathogenesis of muscle injury in these patients has not been fully clarified. A muscle biopsy from a symptomatic MM patient treated with bortezomib revealed abnormalities compatible with a metabolic myopathy characterized on morphological grounds by the accumulation of lipid droplets in several muscle fibers and by mitochondrial alterations consisting of swelling and cristae loss at ultrastructural examination [155]. These pathological findings were reproduced in an in vitro model, indeed, primary human myoblasts exposed to bortezomib within the dose range found in the plasma of treated patients presented excessive storage of lipid droplets together with structural and functional mitochondrial abnormalities, and without being affected by cytotoxic effects of the drug [155]. In line with one of the main mechanisms of cardio- and neurotoxicity induced by bortezomib [76,78,79,80,81,137,156], in vitro findings were consistent with the ability of the drug to impair the mitochondria whose dysfunction may lead to an increase in intracellular lipid content in skeletal muscle [155]. Myogenin, a muscle-specific transcription factor [157], has also been suggested as a possible intracellular target for bortezomib-induced muscle toxicity. It has been observed that the treatment of C2C12 mouse myoblast cell line with bortezomib at clinically relevant doses led to myogenin down-regulation with the consequent inhibition of myotubes formation and cell cycle arrest at the G2/M phase [158].

**Figure 3:**
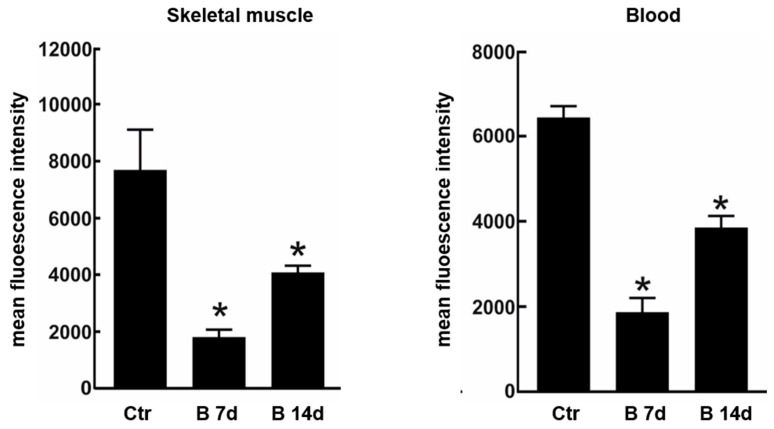
Chymotrypsin-like proteasome activity in skeletal muscle and blood of control mice and mice treated for 7 or 14 days with bortezomib. Chymotrypsin-like activity decreases significantly in skeletal muscle and blood of treated mice compared to control group. The graph presents mean fluorescence intensity ± SD; *n* = 3; * *p* < 0.05 vs controls in one-way Anova with Dunnet’s post hoc test.

**Figure 4:**
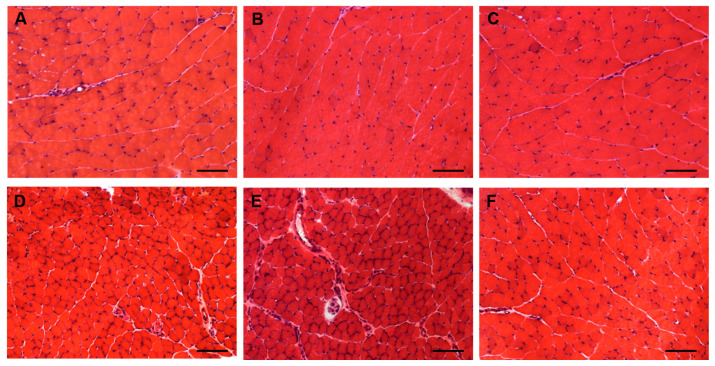
Light microscopy of gastrocnemius (**A**–**C**) and tibialis anterior (**D**–**F**) muscles from control mice (**A**,**D**) and mice treated for 7 (**B**,**E**) or 14 days (**C**,**F**) with bortezomib. H&E stain shows no morphological abnormalities in control mice and in mice treated with bortezomib. Images were obtained with obj ×20. Bars: 100 μm.

**Figure 5:**
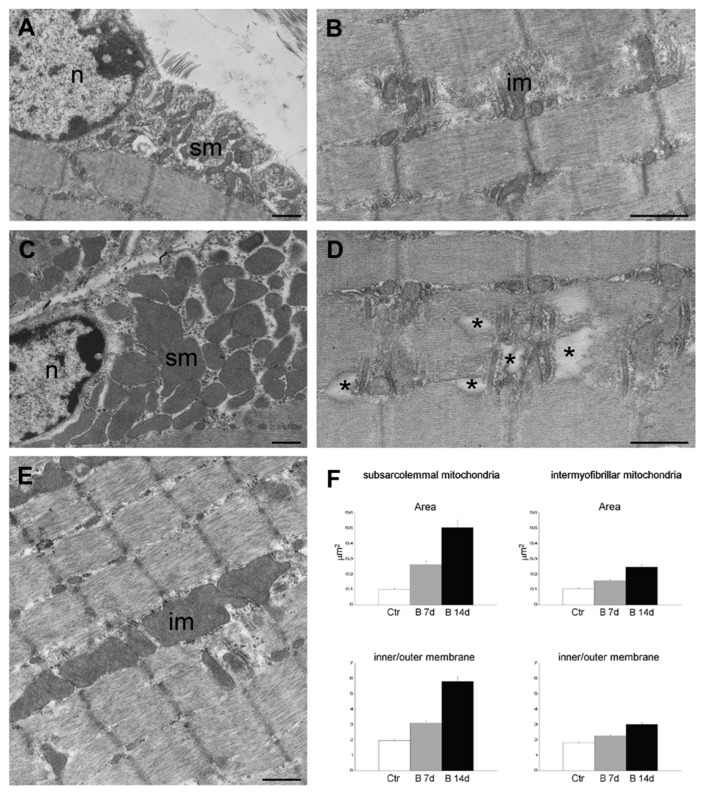
Transmission electron micrographs of gastrocnemius muscles from control mice (**A**,**B**) and mice treated for 14 days with bortezomib (**C**–**E**). Note the large-sized subsarcolemmal (sm) and intermyofibrillar (im) mitochondria in C and E compared to the mitochondria in A and B, and the sarcoplasmic enlargements in D (asterisks). Nucleus (n). Bars: 500 nm. (**F**) Histograms in F show the mean ± SE values of sectional area and inner/outer membrane ratio of subsarcolemmal and intermyofibrillar mitochondria in gastrocnemius muscles from control mice and mice treated for 7 or 14 days with bortezomib. All values are significantly different from each other.

Due to the possible clinical relevance of bortezomib-induced muscle toxicity, our group developed an experimental mouse model to further elucidate the mechanisms responsible for muscle damage. Adult female mice were treated with bortezomib at a dose of 1 mg/kg twice weekly for 7 or 14 days and morphological studies including light microscopy and transmission electron microscopy were performed on skeletal muscle tissue (see File S1 (Supplementary Materials) for the detail description of Materials and Methods). One week after the administration of bortezomib, chymotrypsin-like activity of the proteasome was markedly reduced in blood and skeletal muscle of treated mice compared to the control group (Figure 3). After 14 days of treatment, the chymotrypsin-like activity partially recovered but still was significantly lower in blood as well as in the muscle tissue of mice receiving the drug than in control mice (Figure 3). Histological analysis did not show morphological changes of vastus lateralis, gastrocnemius, and tibialis anterioris muscles in mice exposed to bortezomib for one or two weeks (Figure 4). Neither necrotic or regenerating muscle fibers nor increased perimysial or endomysial connective tissue was observed in hematoxylin and eosin (H&E) and modified Gomori trichrome (Figure 4). All other histochemical stainings including nicotinamide adenine dinucleotide-tetrazolium reductase (NADH-TR), cytochrome c oxidase (COX), succinate dehydrogenase (SDH), Periodic acid Schiff (PAS) and Sudan black were normal. The ultrastructural analysis of muscles obtained from bortezomib-treated mice, however, revealed morphological abnormalities of mitochondria, which were enlarged and had a significantly higher cristae density, more commonly in the subsarcolemmal compartment (Figure 5). Moreover, the lateral sacs of sarcoplasmic reticulum were in various stages of dilation but retained their triadic structure (Figure 5). All these changes were more evident in mice after 14 days of treatment. Neither storage of lipid droplets nor morphological evidence of apoptosis or necrosis was observed in any muscle samples. These findings indicate that bortezomib may exert a toxic effect on mitochondria in skeletal muscle fibers in healthy C57BL/6 mice, suggesting that “mitotoxicity” could represent a common mechanism responsible for the toxicity induced by the PI in the different tissues, including peripheral nerve and heart. Further studies are needed to clarify whether the changes of mitochondrial morphology observed in muscles of bortezomib-treated mice result in their functional impairment and to identify the exact intracellular targets of bortezomib in skeletal muscle.

## 6. Conclusions

Bortezomib has shown remarkable clinical benefits in the treatment of MM but is associated with various adverse events that may lead to early discontinuation of the therapy. Despite peripheral neuropathy is recognized as the main non-hematologic dose-limiting adverse effect, cardiovascular and muscular complications are relatively common and potentially reversible. Therefore, their identification and management are crucial to provide an optimal therapeutic strategy and improve long-term outcomes. Patients treated with bortezomib should undergo cardiac surveillance and be carefully monitored by a neurologist for neuromuscular signs and/or symptoms.

In vivo and in vitro studies have documented a significant alteration of the morphology and/or function of mitochondria in peripheral nerves as well as in cardiac and skeletal muscle, suggesting that they might be a common target of bortezomib toxicity in these tissues. The mechanisms underlying the drug-induced mitochondrial defects are partially known, hampering the development of effective therapeutic strategies able to prevent or reverse them, without interfering with the anti-tumor activity. Modulators of oxidative phosphorylation, ROS scavengers, compounds that boost endogenous antioxidant responses, and drugs preventing the mitochondrial p53 accumulation or able to modulate the mitochondrial metabolism may be suitable for managing the effects of bortezomib on mitochondria [159]. However, single small-sized studies that evaluated the efficacy of acetyl-L-carnitine, methylcobalamin, and a nutraceutical compound (docosahexaenoic acid, α-lipoic acid, vitamin C, and vitamin E) for the prevention of bortezomib-related peripheral neuropathy do not allow to draw firm conclusions on their therapeutic benefits [78,160,161]. Hopefully, available experimental models will help us to further clarify the pathophysiology of mitochondrial toxicity induced by bortezomib, providing valuable information for possible strategies aimed at overcoming these effects. 

## Supplementary Materials

The following are available online at https://www.mdpi.com/2072-6694/12/9/2540/s1, File S1: The detail description of Materials and Methods.

## References

[1] Rajkumar S.V., Dimopoulos M.A., Palumbo A., Blade J., Merlini G., Mateos M.-V., Kumar S., Hillengass J., Kastritis E., Richardson P. (2014). International Myeloma Working Group updated criteria for the diagnosis of multiple myeloma. Lancet Oncol..

[2] Palumbo A., Anderson K. (2011). Multiple Myeloma. N. Engl. J. Med..

[3] Landgren O., Kyle R.A., Pfeiffer R.M., Katzmann J.A., Caporaso N.E., Hayes R.B., Dispenzieri A., Kumar S., Clark R.J., Baris D. (2009). Monoclonal gammopathy of undetermined significance (MGUS) consistently precedes multiple myeloma: A prospective study. Blood.

[4] Kumar S.K., Rajkumar V., Kyle R.A., van Duin M., Sonneveld P., Mateos M.-V., Gay F., Anderson K.C. (2017). Multiple myeloma. Nat. Rev. Dis. Primer.

[5] Howlader N., Noone A.M., Krapcho M., Miller D., Brest A., Yu M., Ruhl J., Tatalovich Z., Mariotto A., Lewis D.R. (2020). SEER Cancer Statistics Review, 1975–2017.

[6] Kumar S.K., Dispenzieri A., Lacy M.Q., Gertz M.A., Buadi F.K., Pandey S., Kapoor P., Dingli D., Hayman S.R., Leung N. (2014). Continued improvement in survival in multiple myeloma: Changes in early mortality and outcomes in older patients. Leukemia.

[7] Gandolfi S., Laubach J.P., Hideshima T., Chauhan D., Anderson K.C., Richardson P.G. (2017). The proteasome and proteasome inhibitors in multiple myeloma. Cancer Metastasis Rev..

[8] Bird S.A., Boyd K. (2019). Multiple myeloma: An overview of management. Palliat. Care Soc. Pract..

[9] Pinto V., Bergantim R., Caires H.R., Seca H., Guimarães J.E., Vasconcelos M.H. (2020). Multiple Myeloma: Available Therapies and Causes of Drug Resistance. Cancers.

[10] Sherman D.J., Li J. (2020). Proteasome Inhibitors: Harnessing Proteostasis to Combat Disease. Molecules.

[11] Walter P., Ron D. (2011). The Unfolded Protein Response: From Stress Pathway to Homeostatic Regulation. Science.

[12] Ri M. (2016). Endoplasmic-reticulum stress pathway-associated mechanisms of action of proteasome inhibitors in multiple myeloma. Int. J. Hematol..

[13] Adams J. (2004). The proteasome: A suitable antineoplastic target. Nat. Rev. Cancer.

[14] Mitsiades N., Mitsiades C.S., Richardson P.G., Poulaki V., Tai Y.-T., Chauhan D., Fanourakis G., Gu X., Bailey C., Joseph M. (2003). The proteasome inhibitor PS-341 potentiates sensitivity of multiple myeloma cells to conventional chemotherapeutic agents: Therapeutic applications. Blood.

[15] Mateos M.-V., Ludwig H., Bazarbachi A., Beksac M., Bladé J., Boccadoro M., Cavo M., Delforge M., Dimopoulos M.A., Facon T. (2019). Insights on Multiple Myeloma Treatment Strategies. HemaSphere.

[16] Kumar S.K., Callander N.S., Hillengass J., Liedtke M., Baljevic M., Campagnaro E., Castillo J.J., Chandler J.C., Cornell R.F., Costello C. (2019). NCCN Guidelines Insights: Multiple Myeloma, Version 1.2020. J. Natl. Compr. Cancer Netw..

[17] Rajkumar S.V., Kumar S. (2016). Multiple Myeloma: Diagnosis and Treatment. Mayo Clin. Proc..

[18] Manasanch E.E., Orlowski R.Z. (2017). Proteasome inhibitors in cancer therapy. Nat. Rev. Clin. Oncol..

[19] Groll M., Berkers C.R., Ploegh H.L., Ovaa H. (2006). Crystal Structure of the Boronic Acid-Based Proteasome Inhibitor Bortezomib in Complex with the Yeast 20S Proteasome. Structure.

[20] Barrio S., Stühmer T., Da-Viá M., Barrio-Garcia C., Lehners N., Besse A., Cuenca I., Garitano-Trojaola A., Fink S., Leich E. (2019). Spectrum and functional validation of PSMB5 mutations in multiple myeloma. Leukemia.

[21] Allmeroth K., Horn M., Kroef V., Miethe S., Müller R.-U., Denzel M.S. (2020). Bortezomib resistance mutations in PSMB5 determine response to second-generation proteasome inhibitors in multiple myeloma. Leukemia.

[22] Lü S., Wang J. (2013). The resistance mechanisms of proteasome inhibitor bortezomib. Biomark. Res..

[23] Oakes S.A., Papa F.R. (2015). The Role of Endoplasmic Reticulum Stress in Human Pathology. Annu. Rev. Pathol. Mech. Dis..

[24] Ito S. (2020). Proteasome Inhibitors for the Treatment of Multiple Myeloma. Cancers.

[25] Li Z.-W., Chen H., Campbell R.A., Bonavida B., Berenson J.R. (2008). NF-kB in the pathogenesis and treatment of multiple myeloma. Curr. Opin. Hematol..

[26] Hideshima T., Mitsiades C., Akiyama M., Hayashi T., Chauhan D., Richardson P., Schlossman R., Podar K., Munshi N.C., Mitsiades N. (2003). Molecular mechanisms mediating antimyeloma activity of proteasome inhibitor PS-341. Blood.

[27] Rajkumar S.V., Richardson P.G., Hideshima T., Anderson K.C. (2005). Proteasome Inhibition As a Novel Therapeutic Target in Human Cancer. J. Clin. Oncol..

[28] Tan C.R.C., Abdul-Majeed S., Cael B., Barta S.K. (2019). Clinical Pharmacokinetics and Pharmacodynamics of Bortezomib. Clin. Pharmacokinet..

[29] Richardson P.G., Barlogie B., Berenson J., Singhal S., Jagannath S., Irwin D., Rajkumar S.V., Srkalovic G., Alsina M., Alexanian R. (2003). A Phase 2 Study of Bortezomib in Relapsed, Refractory Myeloma. N. Engl. J. Med..

[30] Richardson P.G., Sonneveld P., Schuster M.W., Irwin D., Stadtmauer E.A., Facon T., Harousseau J.-L., Ben-Yehuda D., Lonial S., Goldschmidt H. (2005). Bortezomib or High-Dose Dexamethasone for Relapsed Multiple Myeloma. N. Engl. J. Med..

[31] Argyriou A.A., Iconomou G., Kalofonos H.P. (2008). Bortezomib-induced peripheral neuropathy in multiple myeloma: A comprehensive review of the literature. Blood.

[32] Song X., Wilson K.L., Kagan J., Panjabi S. (2019). Cost of peripheral neuropathy in patients receiving treatment for multiple myeloma: A US administrative claims analysis. Ther. Adv. Hematol..

[33] Argyriou A.A., Cavaletti G., Bruna J., Kyritsis A.P., Kalofonos H.P. (2014). Bortezomib-induced peripheral neurotoxicity: An update. Arch. Toxicol..

[34] Giannoccaro M.P., Donadio V., Gomis Pèrez C., Borsini W., Di Stasi V., Liguori R. (2011). Somatic and autonomic small fiber neuropathy induced by bortezomib therapy: An immunofluorescence study. Neurol. Sci..

[35] Mele G., Coppi M.R., Melpignano A., Quarta G. (2016). Paralytic ileus following “subcutaneous bortezomib” therapy: Focus on the clinical emergency—Report of two cases. Clin. Exp. Med..

[36] Shah M.H. (2004). Phase II Study of the Proteasome Inhibitor Bortezomib (PS-341) in Patients with Metastatic Neuroendocrine Tumors. Clin. Cancer Res..

[37] Stratogianni A., Tosch M., Schlemmer H., Weis J., Katona I., Isenmann S., Haensch C.A. (2012). Bortezomib-induced severe autonomic neuropathy. Clin. Auton. Res..

[38] Mauermann M.L., Blumenreich M.S., Dispenzieri A., Staff N.P. (2012). A case of peripheral nerve microvasculitis associated with multiple myeloma and bortezomib treatment. Muscle Nerve.

[39] Chaudhry V., Cornblath D.R., Polydefkis M., Ferguson A., Borrello I. (2008). Characteristics of bortezomib- and thalidomide-induced peripheral neuropathy. J. Peripher. Nerv. Syst..

[40] Ravaglia S., Corso A., Piccolo G., Lozza A., Alfonsi E., Mangiacavalli S., Varettoni M., Zappasodi P., Moglia A., Lazzarino M. (2008). Immune-mediated neuropathies in myeloma patients treated with bortezomib. Clin. Neurophysiol..

[41] Thawani S.P., Tanji K., De Sousa E.A., Weimer L.H., Brannagan T.H. (2015). Bortezomib-Associated Demyelinating Neuropathy—Clinical and Pathologic Features. J. Clin. Neuromuscul. Dis..

[42] Filosto M., Rossi G., Pelizzari A.M., Buzio S., Tentorio M., Broglio L., Mancuso M., Rinaldi M., Scarpelli M., Padovani A. (2007). A high-dose bortezomib neuropathy with sensory ataxia and myelin involvement. J. Neurol. Sci..

[43] Singh M., Thomas V.M., Mulay S. (2020). Bortezomib-induced motor neuropathy: A case report. J. Oncol. Pharm. Pract..

[44] Gupta S., Pagliuca A., Devereux S., Mufti G.J., Schey S. (2006). Life-threatening motor neurotoxicity in association wih bortezomib. Haematologica.

[45] Jeter A., Kang Y. (2012). Immune modulation therapy in the management of bortezomib-induced peripheral neuropathy. Exp. Hematol. Oncol..

[46] Schmitt S., Goldschmidt H., Storch-Hagenlocher B., Pham M., Fingerle-Rowson G., Ho A.D., Neben K. (2011). Inflammatory autoimmune neuropathy, presumably induced by bortezomib, in a patient suffering from multiple myeloma. Int. J. Hematol..

[47] Saifee T.A., Elliott K.J., Lunn M.P., Blake J., Reilly M.M., Rabin N., Yong K.L., D’Sa S., Brandner S. (2010). Bortezomib-induced inflammatory neuropathy. J. Peripher. Nerv. Syst..

[48] Li T., Timmins H.C., King T., Kiernan M.C., Goldstein D., Park S.B. (2020). Characteristics and risk factors of bortezomib induced peripheral neuropathy: A systematic review of phase III trials. Hematol. Oncol..

[49] Velasco R., Alberti P., Bruna J., Psimaras D., Argyriou A.A. (2019). Bortezomib and other proteosome inhibitors—Induced peripheral neurotoxicity: From pathogenesis to treatment. J. Peripher. Nerv. Syst..

[50] Richardson P.G., Briemberg H., Jagannath S., Wen P.Y., Barlogie B., Berenson J., Singhal S., Siegel D.S., Irwin D., Schuster M. (2006). Frequency, Characteristics, and Reversibility of Peripheral Neuropathy During Treatment of Advanced Multiple Myeloma With Bortezomib. J. Clin. Oncol..

[51] Knopf K.B., Duh M.S., Lafeuille M.-H., Gravel J., Lefebvre P., Niculescu L., Ba-Mancini A., Ma E., Shi H., Comenzo R.L. (2014). Meta-Analysis of the Efficacy and Safety of Bortezomib Re-Treatment in Patients With Multiple Myeloma. Clin. Lymphoma Myeloma Leuk..

[52] Vidisheva A.P., Wang J., Spektor T.M., Bitran J.D., Lutzky J., Tabbara I.A., Ye J.Z., Ailawadhi S., Stampleman L.V., Steis R.G. (2017). Safety of BTZ retreatment for patients with low-grade peripheral neuropathy during the initial treatment. Support. Care Cancer.

[53] Bringhen S., Larocca A., Rossi D., Cavalli M., Genuardi M., Ria R., Gentili S., Patriarca F., Nozzoli C., Levi A. (2010). Efficacy and safety of once-weekly bortezomib in multiple myeloma patients. Blood.

[54] Ye Z., Chen J., Xuan Z., Yang W., Chen J. (2019). Subcutaneous bortezomib might be standard of care for patients with multiple myeloma: A systematic review and meta-analysis. Drug Des. Devel. Ther..

[55] Mu S., Ai L., Qin Y., Hu Y. (2018). Subcutaneous versus Intravenous Bortezomib Administration for Multiple Myeloma Patients: A Meta-analysis. Curr. Med. Sci..

[56] Terpos E., Kleber M., Engelhardt M., Zweegman S., Gay F., Kastritis E., van de Donk N.W.C.J., Bruno B., Sezer O., Broijl A. (2015). European Myeloma Network Guidelines for the Management of Multiple Myeloma-related Complications. Haematologica.

[57] Richardson P.G., Delforge M., Beksac M., Wen P., Jongen J.L., Sezer O., Terpos E., Munshi N., Palumbo A., Rajkumar S.V. (2012). Management of treatment-emergent peripheral neuropathy in multiple myeloma. Leukemia.

[58] Dimopoulos M.A., Mateos M.-V., Richardson P.G., Schlag R., Khuageva N.K., Shpilberg O., Kropff M., Spicka I., Palumbo A., Wu K.L. (2011). Risk factors for, and reversibility of, peripheral neuropathy associated with bortezomib-melphalan-prednisone in newly diagnosed patients with multiple myeloma: Subanalysis of the phase 3 VISTA study: Reversibility of PN with VMP in newly diagnosed MM. Eur. J. Haematol..

[59] Richardson P.G., Sonneveld P., Schuster M.W., Stadtmauer E.A., Facon T., Harousseau J.-L., Ben-Yehuda D., Lonial S., Goldschmidt H., Reece D. (2009). Reversibility of symptomatic peripheral neuropathy with bortezomib in the phase III APEX trial in relapsed multiple myeloma: Impact of a dose-modification guideline. Br. J. Haematol..

[60] Boyette-Davis J.A., Cata J.P., Zhang H., Driver L.C., Wendelschafer-Crabb G., Kennedy W.R., Dougherty P.M. (2011). Follow-Up Psychophysical Studies in Bortezomib-Related Chemoneuropathy Patients. J. Pain.

[61] Ibrahim E.Y., Ehrlich B.E. (2020). Prevention of chemotherapy-induced peripheral neuropathy: A review of recent findings. Crit. Rev. Oncol. Hematol..

[62] Lanzani F., Mattavelli L., Frigeni B., Rossini F., Cammarota S., Petrò D., Jann S., Cavaletti G. (2008). Role of a pre-existing neuropathy on the course of bortezomib-induced peripheral neurotoxicity. J. Peripher. Nerv. Syst..

[63] Badros A., Goloubeva O., Dalal J.S., Can I., Thompson J., Rapoport A.P., Heyman M., Akpek G., Fenton R.G. (2007). Neurotoxicity of bortezomib therapy in multiple myeloma: A single-center experience and review of the literature. Cancer.

[64] Richardson P.G., Xie W., Mitsiades C., Chanan-Khan A.A., Lonial S., Hassoun H., Avigan D.E., Oaklander A.L., Kuter D.J., Wen P.Y. (2009). Single-Agent Bortezomib in Previously Untreated Multiple Myeloma: Efficacy, Characterization of Peripheral Neuropathy, and Molecular Correlations With Response and Neuropathy. J. Clin. Oncol..

[65] Corso A., Mangiacavalli S., Varettoni M., Pascutto C., Zappasodi P., Lazzarino M. (2010). Bortezomib-induced peripheral neuropathy in multiple myeloma: A comparison between previously treated and untreated patients. Leuk. Res..

[66] Mateos M.-V., Hernández J.-M., Hernández M.-T., Gutiérrez N.-C., Palomera L., Fuertes M., Díaz-Mediavilla J., Lahuerta J.-J., de la Rubia J., Terol M.-J. (2006). Bortezomib plus melphalan and prednisone in elderly untreated patients with multiple myeloma: Results of a multicenter phase 1/2 study. Blood.

[67] Casafont I., Berciano M.T., Lafarga M. (2010). Bortezomib Induces the Formation of Nuclear poly(A) RNA Granules Enriched in Sam68 and PABPN1 in Sensory Ganglia Neurons. Neurotox. Res..

[68] Carozzi V.A., Canta A., Oggioni N., Sala B., Chiorazzi A., Meregalli C., Bossi M., Marmiroli P., Cavaletti G. (2010). Neurophysiological and neuropathological characterization of new murine models of chemotherapy-induced chronic peripheral neuropathies. Exp. Neurol..

[69] Cata J.P., Weng H.-R., Burton A.W., Villareal H., Giralt S., Dougherty P.M. (2007). Quantitative Sensory Findings in Patients With Bortezomib-Induced Pain. J. Pain.

[70] Terkelsen A.J., Karlsson P., Lauria G., Freeman R., Finnerup N.B., Jensen T.S. (2017). The diagnostic challenge of small fibre neuropathy: Clinical presentations, evaluations, and causes. Lancet Neurol..

[71] Meregalli C., Canta A., Carozzi V.A., Chiorazzi A., Oggioni N., Gilardini A., Ceresa C., Avezza F., Crippa L., Marmiroli P. (2010). Bortezomib-induced painful neuropathy in rats: A behavioral, neurophysiological and pathological study in rats. Eur. J. Pain.

[72] Carozzi V.A., Renn C.L., Bardini M., Fazio G., Chiorazzi A., Meregalli C., Oggioni N., Shanks K., Quartu M., Serra M.P. (2013). Bortezomib-Induced Painful Peripheral Neuropathy: An Electrophysiological, Behavioral, Morphological and Mechanistic Study in the Mouse. PLoS ONE.

[73] Bruna J., Udina E., Alé A., Vilches J.J., Vynckier A., Monbaliu J., Silverman L., Navarro X. (2010). Neurophysiological, histological and immunohistochemical characterization of bortezomib-induced neuropathy in mice. Exp. Neurol..

[74] Bruna J., Alé A., Velasco R., Jaramillo J., Navarro X., Udina E. (2011). Evaluation of pre-existing neuropathy and bortezomib retreatment as risk factors to develop severe neuropathy in a mouse model. J. Peripher. Nerv. Syst..

[75] Shin Y.K., Jang S.Y., Lee H.K., Jung J., Suh D.J., Seo S.-Y., Park H.T. (2010). Pathological adaptive responses of Schwann cells to endoplasmic reticulum stress in bortezomib-induced peripheral neuropathy. Glia.

[76] Cavaletti G., Gilardini A., Canta A., Rigamonti L., Rodriguez-Menendez V., Ceresa C., Marmiroli P., Bossi M., Oggioni N., D’Incalci M. (2007). Bortezomib-induced peripheral neurotoxicity: A neurophysiological and pathological study in the rat. Exp. Neurol..

[77] Gilardini A., Avila R.L., Oggioni N., Rodriguez-Menendez V., Bossi M., Canta A., Cavaletti G., Kirschner D.A. (2012). Myelin structure is unaltered in chemotherapy-induced peripheral neuropathy. NeuroToxicology.

[78] Zheng H., Xiao W.H., Bennett G.J. (2012). Mitotoxicity and bortezomib-induced chronic painful peripheral neuropathy. Exp. Neurol..

[79] Jannuzzi A.T., Arslan S., Yilmaz A.M., Sari G., Beklen H., Méndez L., Fedorova M., Arga K.Y., Karademir Yilmaz B., Alpertunga B. (2020). Higher proteotoxic stress rather than mitochondrial damage is involved in higher neurotoxicity of bortezomib compared to carfilzomib. Redox Biol..

[80] Landowski T.H., Megli C.J., Nullmeyer K.D., Lynch R.M., Dorr R.T. (2005). Mitochondrial-Mediated Disregulation of Ca^2+^ Is a Critical Determinant of Velcade (PS-341/Bortezomib) Cytotoxicity in Myeloma Cell Lines. Cancer Res..

[81] Sullivan P.G., Dragicevic N.B., Deng J.-H., Bai Y., Dimayuga E., Ding Q., Chen Q., Bruce-Keller A.J., Keller J.N. (2004). Proteasome Inhibition Alters Neural Mitochondrial Homeostasis and Mitochondria Turnover. J. Biol. Chem..

[82] Nasu S., Misawa S., Nakaseko C., Shibuya K., Isose S., Sekiguchi Y., Mitsuma S., Ohmori S., Iwai Y., Beppu M. (2014). Bortezomib-induced neuropathy: Axonal membrane depolarization precedes development of neuropathy. Clin. Neurophysiol..

[83] Xiao W.H., Bennett G.J. (2012). Effects of mitochondrial poisons on the neuropathic pain produced by the chemotherapeutic agents, paclitaxel and oxaliplatin. Pain.

[84] Xiao W.H., Zheng H., Zheng F.Y., Nuydens R., Meert T.F., Bennett G.J. (2011). Mitochondrial abnormality in sensory, but not motor, axons in paclitaxel-evoked painful peripheral neuropathy in the rat. Neuroscience.

[85] Jordan M.A., Wilson L. (2004). Microtubules as a target for anticancer drugs. Nat. Rev. Cancer.

[86] Klimaschewski L., Hausott B., Ingorokva S., Pfaller K. (2006). Constitutively expressed catalytic proteasomal subunits are up-regulated during neuronal differentiation and required for axon initiation, elongation and maintenance. J. Neurochem..

[87] Laser H., Mack T.G.A., Wagner D., Coleman M.P. (2003). Proteasome inhibition arrests neurite outgrowth and causes "dying-back" degeneration in primary culture. J. Neurosci. Res..

[88] Poruchynsky M.S., Sackett D.L., Robey R.W., Ward Y., Annunziata C., Fojo T. (2008). Proteasome inhibitors increase tubulin polymerization and stabilization in tissue culture cells: A possible mechanism contributing to peripheral neuropathy and cellular toxicity following proteasome inhibition. Cell Cycle.

[89] Meregalli C., Chiorazzi A., Carozzi V.A., Canta A., Sala B., Colombo M., Oggioni N., Ceresa C., Foudah D., La Russa F. (2014). Evaluation of tubulin polymerization and chronic inhibition of proteasome as citotoxicity mechanisms in bortezomib-induced peripheral neuropathy. Cell Cycle.

[90] Alé A., Bruna J., Herrando M., Navarro X., Udina E. (2015). Toxic Effects of Bortezomib on Primary Sensory Neurons and Schwann Cells of Adult Mice. Neurotox. Res..

[91] Staff N.P., Podratz J.L., Grassner L., Bader M., Paz J., Knight A.M., Loprinzi C.L., Trushina E., Windebank A.J. (2013). Bortezomib alters microtubule polymerization and axonal transport in rat dorsal root ganglion neurons. NeuroToxicology.

[92] Karademir B., Sari G., Jannuzzi A.T., Musunuri S., Wicher G., Grune T., Mi J., Hacioglu-Bay H., Forsberg-Nilsson K., Bergquist J. (2018). Proteomic approach for understanding milder neurotoxicity of Carfilzomib against Bortezomib. Sci. Rep..

[93] Palanca A., Casafont I., Berciano M.T., Lafarga M. (2014). Proteasome inhibition induces DNA damage and reorganizes nuclear architecture and protein synthesis machinery in sensory ganglion neurons. Cell. Mol. Life Sci..

[94] Stockstill K., Doyle T.M., Yan X., Chen Z., Janes K., Little J.W., Braden K., Lauro F., Giancotti L.A., Harada C.M. (2018). Dysregulation of sphingolipid metabolism contributes to bortezomib-induced neuropathic pain. J. Exp. Med..

[95] Mangiacavalli S., Corso A., De Amici M., Varettoni M., Alfonsi E., Lozza A., Lazzarino M. (2010). Emergent T-helper 2 profile with high interleukin-6 levels correlates with the appearance of bortezomib-induced neuropathic pain: Correspondence. Br. J. Haematol..

[96] Hung A.L., Lim M., Doshi T.L. (2017). Targeting cytokines for treatment of neuropathic pain. Scand. J. Pain.

[97] Stemkowski P.L., Smith P.A. (2012). Sensory Neurons, Ion Channels, Inflammation and the Onset of Neuropathic Pain. Can. J. Neurol. Sci..

[98] Myers R.R., Shubayev V.I. (2011). The ology of neuropathy: An integrative review of the role of neuroinflammation and TNF-α axonal transport in neuropathic pain. J. Peripher. Nerv. Syst..

[99] Leung L., Cahill C.M. (2010). TNF-α and neuropathic pain—A review. J. Neuroinflamm..

[100] Alé A., Bruna J., Morell M., van de Velde H., Monbaliu J., Navarro X., Udina E. (2014). Treatment with anti-TNF alpha protects against the neuropathy induced by the proteasome inhibitor bortezomib in a mouse model. Exp. Neurol..

[101] Zhang J., Su Y.-M., Li D., Cui Y., Huang Z.-Z., Wei J.-Y., Xue Z., Pang R.-P., Liu X.-G., Xin W.-J. (2014). TNF-α-mediated JNK activation in the dorsal root ganglion neurons contributes to Bortezomib-induced peripheral neuropathy. Brain. Behav. Immun..

[102] Li Z.-Y., Zhang Y.-P., Zhang J., Zhang S.-B., Li D., Huang Z.-Z., Xin W.-J. (2016). The possible involvement of JNK activation in the spinal dorsal horn in bortezomib-induced allodynia: The role of TNF-α and IL-1β. J. Anesth..

[103] Li C., Deng T., Shang Z., Wang D., Xiao Y. (2018). Blocking TRPA1 and TNF-α Signal Improves Bortezomib-Induced Neuropathic Pain. Cell. Physiol. Biochem..

[104] Chiorazzi A., Canta A., Meregalli C., Carozzi V., Sala B., Oggioni N., Monbaliu J. (2013). Antibody Against Tumor Necrosis Factor-α Reduces Bortezomib-induced Allodynia in a Rat Model. Anticancer Res..

[105] Hideshima T., Richardson P., Chauhan D., Palombella V.J., Elliott P.J., Adams J., Anderson K.C. (2001). The Proteasome Inhibitor PS-341 Inhibits Growth, Induces Apoptosis, and Overcomes Drug Resistance in Human Multiple Myeloma Cells. Cancer Res..

[106] Karin M. (2006). Nuclear factor-κB in cancer development and progression. Nature.

[107] Alé A., Bruna J., Calls A., Karamita M., Haralambous S., Probert L., Navarro X., Udina E. (2016). Inhibition of the neuronal NFκB pathway attenuates bortezomib-induced neuropathy in a mouse model. NeuroToxicology.

[108] Cole D.C., Frishman W.H. (2018). Cardiovascular Complications of Proteasome Inhibitors Used in Multiple Myeloma. Cardiol. Rev..

[109] Kistler K.D., Kalman J., Sahni G., Murphy B., Werther W., Rajangam K., Chari A. (2017). Incidence and Risk of Cardiac Events in Patients With Previously Treated Multiple Myeloma Versus Matched Patients Without Multiple Myeloma: An Observational, Retrospective, Cohort Study. Clin. Lymphoma Myeloma Leuk..

[110] Orciuolo E., Buda G., Cecconi N., Galimberti S., Versari D., Cervetti G., Salvetti A., Petrini M. (2007). Unexpected cardiotoxicity in haematological bortezomib treated patients. Br. J. Haematol..

[111] Gupta A., Pandey A., Sethi S. (2012). Bortezomib-Induced Congestive Cardiac Failure in a Patient with Multiple Myeloma. Cardiovasc. Toxicol..

[112] Bockorny M., Chakravarty S., Schulman P., Bockorny B., Bona R. (2012). Severe Heart Failure after Bortezomib Treatment in a Patient with Multiple Myeloma: A Case Report and Review of the Literature. Acta Haematol..

[113] Meseeha M.G., Kolade V.O., Attia M.N. (2015). Partially reversible bortezomib-induced cardiotoxicity: An unusual cause of acute cardiomyopathy. J. Community Hosp. Intern. Med. Perspect..

[114] Voortman J., Giaccone G. (2006). Severe reversible cardiac failure after bortezomib treatment combined with chemotherapy in a non-small cell lung cancer patient: A case report. BMC Cancer.

[115] Honton B., Despas F., Dumonteil N., Rouvellat C., Roussel M., Carrie D., Galinier M., Montastruc J.L., Pathak A. (2014). Bortezomib and heart failure: Case-report and review of the French Pharmacovigilance database. Fundam. Clin. Pharmacol..

[116] Hacihanefioglu A., Tarkun P., Gonullu E. (2008). Acute severe cardiac failure in a myeloma patient due to proteasome inhibitor bortezomib. Int. J. Hematol..

[117] Jerkins J. (2010). Bortezomib-induced Severe Congestive Heart Failure. Cardiol. Res..

[118] Chakraborty R., Mukkamalla S.K.R., Calderon N. (2013). Bortezomib induced reversible left ventricular systolic dysfunction: A case report and review of literature. BJMP.

[119] Foley P.W., Hamilton M.S., Leyva F. (2010). Myocardial scarring following chemotherapy for multiple myeloma detected using late gadolinium hyperenhancement cardiovascular magnetic resonance. J. Cardiovasc. Med..

[120] Subedi A., Sharma L.R., Shah B.K. (2014). Bortezomib-induced acute congestive heart failure: A case report and review of literature. Ann. Hematol..

[121] Dasanu C.A. (2011). Complete heart block secondary to bortezomib use in multiple myeloma. J. Oncol. Pharm. Pract..

[122] Lee W.-S., Kim D.-H., Shin S.-H., Woo S.-I., Kwan J., Park K.-S., Park S.-D., Yi H.-G., Jeon S.-H. (2011). Complete Atrioventricular Block Secondary to Bortezomib Use in Multiple Myeloma. Yonsei Med. J..

[123] Diwadkar S., Patel A.A., Fradley M.G. (2016). Bortezomib-Induced Complete Heart Block and Myocardial Scar: The Potential Role of Cardiac Biomarkers in Monitoring Cardiotoxicity. Case Rep. Cardiol..

[124] Berenson J.R., Matous J., Swift R.A., Mapes R., Morrison B., Yeh H.S. (2007). A Phase I/II Study of Arsenic Trioxide/Bortezomib/Ascorbic Acid Combination Therapy for the Treatment of Relapsed or Refractory Multiple Myeloma. Clin. Cancer Res..

[125] Takamatsu H., Yamashita T., Kotani T., Sawazaki A., Okumura H., Nakao S. (2010). Ischemic heart disease associated with bortezomib treatment combined with dexamethasone in a patient with multiple myeloma. Int. J. Hematol..

[126] Burkhart T., Keith M.C.L., Lenneman C.A.G., Fernando R.R. (2018). Bortezomib-Induced Cardiac Tamponade in a 49-Year-Old Man. Tex. Heart Inst. J..

[127] Xiao Y., Yin J., Wei J., Shang Z. (2014). Incidence and Risk of Cardiotoxicity Associated with Bortezomib in the Treatment of Cancer: A Systematic Review and Meta-Analysis. PLoS ONE.

[128] Laubach J.P., Moslehi J.J., Francis S.A., San Miguel J.F., Sonneveld P., Orlowski R.Z., Moreau P., Rosiñol L., Faber E.A., Voorhees P. (2017). A retrospective analysis of 3954 patients in phase 2/3 trials of bortezomib for the treatment of multiple myeloma: Towards providing a benchmark for the cardiac safety profile of proteasome inhibition in multiple myeloma. Br. J. Haematol..

[129] Cornell R.F., Ky B., Weiss B.M., Dahm C.N., Gupta D.K., Du L., Carver J.R., Cohen A.D., Engelhardt B.G., Garfall A.L. (2019). Prospective Study of Cardiac Events During Proteasome Inhibitor Therapy for Relapsed Multiple Myeloma. J. Clin. Oncol..

[130] Koulaouzidis G., Lyon A.R. (2017). Proteasome Inhibitors as a Potential Cause of Heart Failure. Heart Fail. Clin..

[131] Shukla S.K., Rafiq K. (2019). Proteasome biology and therapeutics in cardiac diseases. Transl. Res..

[132] Portbury A.L., Ronnebaum S.M., Zungu M., Patterson C., Willis M.S. (2012). Back to your heart: Ubiquitin proteasome system-regulated signal transduction. J. Mol. Cell. Cardiol..

[133] Gilda J.E., Gomes A.V. (2017). Proteasome dysfunction in cardiomyopathies: Proteasome dysfunction in cardiomyopathies. J. Physiol..

[134] Li Y.-F., Wang X. (2011). The role of the proteasome in heart disease. Biochim. Biophys. Acta BBA Gene Regul. Mech..

[135] Hasinoff B.B., Patel D., Wu X. (2017). Molecular Mechanisms of the Cardiotoxicity of the Proteasomal-Targeted Drugs Bortezomib and Carfilzomib. Cardiovasc. Toxicol..

[136] Pokorna Z., Jirkovsky E., Hlavackova M., Jansova H., Jirkovska A., Lencova-Popelova O., Brazdova P., Kubes J., Sotakova-Kasparova D., Mazurova Y. (2019). In vitro and in vivo investigation of cardiotoxicity associated with anticancer proteasome inhibitors and their combination with anthracycline. Clin. Sci..

[137] Nowis D., Mączewski M., Mackiewicz U., Kujawa M., Ratajska A., Wieckowski M.R., Wilczyński G.M., Malinowska M., Bil J., Salwa P. (2010). Cardiotoxicity of the Anticancer Therapeutic Agent Bortezomib. Am. J. Pathol..

[138] Tang M., Li J., Huang W., Su H., Liang Q., Tian Z., Horak K.M., Molkentin J.D., Wang X. (2010). Proteasome functional insufficiency activates the calcineurin–NFAT pathway in cardiomyocytes and promotes maladaptive remodelling of stressed mouse hearts. Cardiovasc. Res..

[139] Carrier L. (2010). Too much of a good thing is bad: Proteasome inhibition induces stressed hearts to fail. Cardiovasc. Res..

[140] Bonuccelli G., Sotgia F., Capozza F., Gazzerro E., Minetti C., Lisanti M.P. (2007). Localized Treatment with a Novel FDA-Approved Proteasome Inhibitor Blocks the Degradation of Dystrophin and Dystrophin-Associated Proteins in mdx Mice. Cell Cycle.

[141] Herrmann J., Saguner A.M., Versari D., Peterson T.E., Chade A., Olson M., Lerman L.O., Lerman A. (2007). Chronic Proteasome Inhibition Contributes to Coronary Atherosclerosis. Circ. Res..

[142] Kisselev A.F., Goldberg A.L. (2001). Proteasome inhibitors: From research tools to drug candidates. Chem. Biol..

[143] Herrmann J., Wohlert C., Saguner A.M., Flores A., Nesbitt L.L., Chade A., Lerman L.O., Lerman A. (2013). Primary proteasome inhibition results in cardiac dysfunction. Eur. J. Heart Fail..

[144] Gavazzoni M., Vizzardi E., Gorga E., Bonadei I., Rossi L., Belotti A., Rossi G., Ribolla R., Metra M., Raddino R. (2018). Mechanism of cardiovascular toxicity by proteasome inhibitors: New paradigm derived from clinical and pre-clinical evidence. Eur. J. Pharmacol..

[145] Mårtensson C.U., Priesnitz C., Song J., Ellenrieder L., Doan K.N., Boos F., Floerchinger A., Zufall N., Oeljeklaus S., Warscheid B. (2019). Mitochondrial protein translocation-associated degradation. Nature.

[146] Yui J.C., Van Keer J., Weiss B.M., Waxman A.J., Palmer M.B., D’Agati V.D., Kastritis E., Dimopoulos M.A., Vij R., Bansal D. (2016). Proteasome inhibitor associated thrombotic microangiopathy: Proteasome inhibitor associated TMA. Am. J. Hematol..

[147] Roccaro A.M., Hideshima T., Raje N., Kumar S., Ishitsuka K., Yasui H., Shiraishi N., Ribatti D., Nico B., Vacca A. (2006). Bortezomib Mediates Antiangiogenesis in Multiple Myeloma via Direct and Indirect Effects on Endothelial Cells. Cancer Res..

[148] Tamura D., Arao T., Tanaka K., Kaneda H., Matsumoto K., Kudo K., Aomatsu K., Fujita Y., Watanabe T., Saijo N. (2010). Bortezomib potentially inhibits cellular growth of vascular endothelial cells through suppression of G2/M transition. Cancer Sci..

[149] Wei Q., Xia Y. (2006). Proteasome Inhibition Down-regulates Endothelial Nitric-oxide Synthase Phosphorylation and Function. J. Biol. Chem..

[150] Hu X.-S., Du C.-Q., Yang L., Yao X.-Y., Hu S.-J. (2010). Proteasome inhibitor MG132 suppresses number and function of endothelial progenitor cells: Involvement of nitric oxide synthase inhibition. Int. J. Mol. Med..

[151] Herrmann J., Edwards W.D., Holmes D.R., Shogren K.L., Lerman L.O., Ciechanover A., Lerman A. (2002). Increased ubiquitin immunoreactivity in unstable atherosclerotic plaques associated with acute coronary syndromes. J. Am. Coll. Cardiol..

[152] Versari D., Herrmann J., Gössl M., Mannheim D., Sattler K., Meyer F.B., Lerman L.O., Lerman A. (2006). Dysregulation of the Ubiquitin-Proteasome System in Human Carotid Atherosclerosis. Arterioscler. Thromb. Vasc. Biol..

[153] Muchtar E., Derudas D., Mauermann M., Liewluck T., Dispenzieri A., Kumar S.K., Dingli D., Lacy M.Q., Buadi F.K., Hayman S.R. (2016). Systemic Immunoglobulin Light Chain Amyloidosis–Associated Myopathy: Presentation, Diagnostic Pitfalls, and Outcome. Mayo Clin. Proc..

[154] Jagannath S., Barlogie B., Berenson J., Siegel D., Irwin D., Richardson P.G., Niesvizky R., Alexanian R., Limentani S.A., Alsina M. (2004). A phase 2 study of two doses of bortezomib in relapsed or refractory myeloma. Br. J. Haematol..

[155] Guglielmi V., Nowis D., Tinelli M., Malatesta M., Paoli L., Marini M., Manganotti P., Sadowski R., Wilczynski G.M., Meneghini V. (2017). Bortezomib-Induced Muscle Toxicity in Multiple Myeloma. J. Neuropathol. Exp. Neurol..

[156] Arastu-Kapur S., Anderl J.L., Kraus M., Parlati F., Shenk K.D., Lee S.J., Muchamuel T., Bennett M.K., Driessen C., Ball A.J. (2011). Nonproteasomal Targets of the Proteasome Inhibitors Bortezomib and Carfilzomib: A Link to Clinical Adverse Events. Clin. Cancer Res..

[157] Zammit P.S. (2017). Function of the myogenic regulatory factors Myf5, MyoD, Myogenin and MRF4 in skeletal muscle, satellite cells and regenerative myogenesis. Semin. Cell Dev. Biol..

[158] Xing S.S., Shen C.C., Godard M.P., Wang J.J., Yue Y.Y., Yang S.T., Zhao Q., Zhang S.B., Wang T.X., Yang X.L. (2014). Bortezomib inhibits C2C12 growth by inducing cell cycle arrest and apoptosis. Biochem. Biophys. Res. Commun..

[159] Jiacheng M., Kavelaars A., Dougherty P.M., Heijnen C.J. (2018). Beyond Symptomatic Relief for Chemotherapy-Induced Peripheral Neuopathy:targeting the source. Cancer.

[160] Maschio M., Zarabla A., Maialetti A., Marchesi F., Giannarelli D., Gumenyuk S., Pisani F., Renzi D., Galiè E., Mengarelli A. (2018). Prevention of Bortezomib-Related Peripheral Neuropathy with Docosahexaenoic acid and α-Lipoic Acid in Patients with Multiple Myeloma: Preliminary Data. Integr. Cancer Ther..

[161] Callander N., Markovina S., Eickhoff J., Hutson P., Campell T., Hematti P., Go R., Hegeman R., Longo W., Williams E. (2014). Acetyl-L-carnitine (ALCAR) for the prevention of chemotherapy-induced peripheral neuropathy in patients with relapsed or refractory multiple myeloma treated with bortezomib, doxorubin and low-dose dexamethasone: A study from the Wisconsin Ocology Network. Cancer Chemother. Pharmacol..

