# Supplementary material for: Non-Hematologic Toxicity of Bortezomib in Multiple Myeloma: The Neuromuscular and Cardiovascular Adverse Effects
Source: Cancers (Basel). 2020 Sep 7;12(9):2540. doi: 10.3390/cancers12092540 (PMC7563977; doi:10.3390/cancers12092540)
Supplement: Supplementary file 1 [file cancers-12-02540-s001.pdf]

Supplementary Materials:

NON-HEMATOLOGIC TOXICITY OF BORTEZOMIB IN MULTIPLE MYELOMA: THE NEUROMUSCULAR AND CARDIOVASCULAR ADVERSE EFFECTS

Elia Pancheri, Valeria Guglielmi, Grzegorz M. Wilczynski, Manuela Malatesta, Paola Tonin, Giuliano Tomelleri, Dominika Nowis, Gaetano Vattemi

Animals and treatment

Female C57Bl/6 mice (12-13 weeks of age) were used in the experiments. Mice were obtained from the Animal House of the Polish Academy of Sciences Medical Research Center (Warsaw, Poland). All *in vivo* experiments were performed following the guidelines approved by the Ethical Committee of the Medical University of Warsaw (approval No 618/2018), based on national laws that are in full agreement with the European Union directive and US National Institutes of Health guidelines on animal experimentation. Mice were injected i.p. with 1 mg/kg of bortezomib (Velcade, Millenium Pharmaceuticals, Cambridge, MA) every 3rd day for 7 or 14 days. Control mice received i.p. injections of 0.9% saline using the same schedule. Each experimental group consisted at least of 3 animals. On the day of tissue collection mice were anesthetized with isoflurane overdose followed by cervical dislocation. Blood was collected by heart puncture into heparinized tubes, spun down at 1000 x g for 10 min, and the pellet was frozen at -80°C for further evaluation. Vastus lateralis, gastrocnemius and tibialis anterior muscles from both lower limbs were carefully excised *en block*. For histological analysis, muscles were snap-frozen in isopentane cooled with liquid nitrogen whereas for proteasome activity studies samples were flash-frozen in liquid nitrogen. For electron microscopy, a small fragment of each collected muscle was fixed in 2.5% glutaraldehyde in 0.1 M phosphate buffer pH 7.3. All snap-frozen samples were immediately stored at -80°C for further evaluation.

Evaluation of proteasome activity

To determine proteasome activity in blood and skeletal muscle frozen samples were decomposed on ice using VDI12 homogenizer (VWR International, Vienna, Austria) in an assay buffer composed of 0.05 M Tris-HCl, pH 7.6 without protease inhibitors. The homogenates were centrifuged for 10 min at 12 000 x g at 4°C and protein concentration in supernatants was estimated using Bio-Rad Bradford's protein assay (BioRad, Hercules, CA). Equal amounts of total protein (20 μg per well) were dispensed into a black 96-well plate (Fluotrac 200, Greiner Bio-One, Monroe, NC). Proteasome activity was determined using 60 μM fluorogenic peptide substrate for chymotrypsin-like proteasome activity (Suc-Leu-Leu-Val-Tyr-AMC, Bachem, Weil am Rhein, Germany) after 30 min incubation of the enzymatic reaction at 37°C. Hydrolysis of the fluorogenic substrate was measured using VictorX4 microplate fluorometer (Perkin-Elmer, Waltham, MA) equipped with 355 nm excitation and 460 nm emission filters. Tissue samples preincubated for 15 min with 10 μM MG132 (Calbiochem, San Diego, CA) for complete abolishing of ChTL proteasome activity served as background.

Histology and histochemistry

Serial 8-μm-thick cryosections were stained with hematoxylin and eosin (H&E), modified Gomori trichrome, ATPase (pH 4.3, 4.6 and 10.4), succinate dehydrogenase (SDH), cytochrome c

oxidase (COX), nicotinamide adenine dinucleotide-tetrazolium reductase (NADH-TR), Periodic acid Schiff (PAS), Sudan black and acid phosphatase.

Transmission electron microscopy

A small fragment of each collected muscle was fixed in 2.5% glutaraldehyde in 0.1 M phosphate buffer, pH 7.3, at 4 °C overnight, post-fixed with 1% osmium tetroxide and 1.5% potassium ferrocyanide at RT for 2 h, dehydrated with acetone and embedded in Epon. Ultrathin sections were stained with uranyl acetate and observed with a Philips Morgagni transmission electron microscope (FEI Company Italia Srl, Milan, Italy) operating at 80 kV and equipped with a Megaview II camera for digital image acquisition. For morphometric analysis, the gastrocnemius muscle of three mice per experimental group was considered: the sectional area and the length of the outer and inner mitochondrial membrane were measured (X28000) in 30 intermyofibrillar and 30 subsarcolemmal mitochondria per animal, and the inner/outer membrane ratio was calculated as an assessment of cristae extension independent of mitochondrial size. Measurements were made by using the Radius software for image acquisition and elaboration implemented in the Philips Morgagni transmission electron microscope.

Statistical analysis

Data were analyzed using Microsoft Excel 2010 and GraphPad Prism 6.0 for Windows (GraphPad Software Inc.) software. Differences in proteasome activity were analyzed for significance by 1-way ANOVA with Dunnet's post hoc test. Significance was defined as $p < 0.05$.
